# The Hygrometric Properties of Glycerine

**Published:** 1879-08

**Authors:** 


### THE HYGROMETRIC PROPERTIES OF GLYCERINE.

A writer in the *Pharmaceutical Journal* gives the following curious and interesting facts in regard to the behavior of glycerine in a very moist atmosphere: The moisture in the form of water collects and floats on its surface, and taking up, or dissolving, a considerable proportion of the subjacent glycerine (probably more than half its own weight) attracts more moisture, which, in turn, exercises its solvent power and acquires a capability of still further absorption. Thus the action goes on, not necessarily, as may be thought, in a constantly decreasing ratio as the water increases in amount, but at an almost uniform rate from week to week. The mixture of glycerine and water is not so actively hygrometric as the glycerine alone, but the combination once effected the action continues with singular uniformity.

The author (Mr. W. Willmott) then proceeds to illustrate this by means of a table, from which he shows that although during a period of four weeks there is an increase of weight from week to week, yet there is a diminution of the increase during the second and following weeks as compared with the first; and this is owing to a lessening of the intensity of absorption by the presence of the water. All this goes on without stirring or disturbing the fluids in any way. If, however, the water be kept stirred into the glycerine instead of being allowed to remain on its surface, there will be no appreciable difference in this increase of weight between the first and following weeks. But at what point is there a pause in this process? Where does it end?

In whatever proportionate quantity water may be added to glycerine, from a single drop upward, absorption will take place in a moisture-laden atmosphere until the proportion reaches three parts by measure of the former to one of the latter. At this point the glycerine, so to speak, gives up the contest and succumbs to the influence which the water exerts in the opposite direction. In this mixture, therefore, namely, three fluid ounces of water to one of glycerine, there will be neither attraction nor evaporation, the weight scarcely varying from week to week either in one direction or the other. If now we conduct our experiments in a moderately dry atmosphere—say in the atmosphere of an ordinary room in which a fire is kept burning during the day—the action will be the same, but to obtain similar results the proportions will be widely different, and, in fact, almost reversed. Instead of three parts of water to one of glycerine, we shall require nearly three parts of glycerine to one of water to reach the neutral point. Where in one case there is absorption and augmentation, in the other there is evaporation and consequent loss.

It is to this hygroscopic character of glycerine and its power to absorb moisture that is due its irritating effect when rubbed on the skin in an undiluted state.

